# Polypropylene Mesh Infection From Surgical Site Infections Caused by Mycobacterium fortuitum

**DOI:** 10.7759/cureus.61263

**Published:** 2024-05-28

**Authors:** Kota Tsuchiya, Nao Hayashi, Goh Ohji, Hiroto Terashi, Shunsuke Sakakibara

**Affiliations:** 1 Department of Plastic Surgery, Seirei Mikatahara General Hospital, Hamamatsu, JPN; 2 Department of Plastic Surgery, Kobe University Graduate School of Medicine, Kobe, JPN; 3 Division of Infectious Diseases, Kobe University Hospital, Kobe, JPN

**Keywords:** hernia mesh, rapidly growing mycobacterium, mycobacterium fortuitum, non-tuberculous mycobacteria, surgical site infection

## Abstract

This report highlights two cases of surgical site infections (SSIs) caused by *Mycobacterium fortuitum* (Mf) following abdominal mesh implantation. The first case involved an 83-year-old male experiencing non-healing erythema and wounds post-operation, which persisted despite multiple treatments, until effective management was achieved with targeted antibiotics after Mf identification. The second case concerned a female patient with a gynecological postoperative hernia, where Mf was quickly detected following SSI onset three weeks after surgery. Prompt mesh removal and appropriate antibiotic treatment led to a rapid and full recovery. These cases emphasize the importance of early detection and intervention in managing Mf infections effectively, illustrating how the timing of diagnosis can significantly influence treatment outcomes.

## Introduction

Surgical site infections (SSIs) account for 20% of hospital-acquired infections [1]. SSIs are implicated in increased rates of hospital readmission, extended hospital stays, elevated healthcare costs, and higher mortality [2], underscoring the importance of SSI prevention. SSIs are typically caused by bacterial skin flora, with Staphylococci and Streptococci being common pathogens [3]. However, recent reports have indicated an increase in SSIs caused by nontuberculous mycobacteria (NTM) [4]. NTM can be challenging to detect with Gram staining and may not respond to standard antibiotic treatments, which can delay diagnosis and treatment if managed as typical SSIs [4]. Although generally considered of low pathogenicity, NTM can cause localized symptoms in immunocompetent individuals and not just in those with compromised immunity (e.g., HIV-positive patients, those undergoing chemotherapy, or those undergoing corticosteroid treatment) [5]. Failure to properly detect and treat these organisms can necessitate prolonged treatment. *Mycobacterium fortuitum* (Mf) has been frequently reported as a causative agent of NTM-related SSIs [6]. Mf is a rapidly growing mycobacterium (RGM) that forms colonies in culture for approximately one week. It belongs to group IV of NTMs classified by Runyon [7]. NTMs are widely distributed in nature, such as in soil and water [8], and cause infections in various organs in humans and animals. The incidence of NTM infections has been increasing in recent years and is beginning to be considered a public health problem [9]. Mf infections are a common cause of extrapulmonary diseases and skin and soft tissue infections [8], which are frequently reported, especially postoperatively [10]. Here, we report two cases of mesh infection due to SSI caused by Mf after abdominal wall reinforcement with polypropylene mesh (hereafter referred to as mesh).

## Case presentation

Case 1

**Figure 1:**
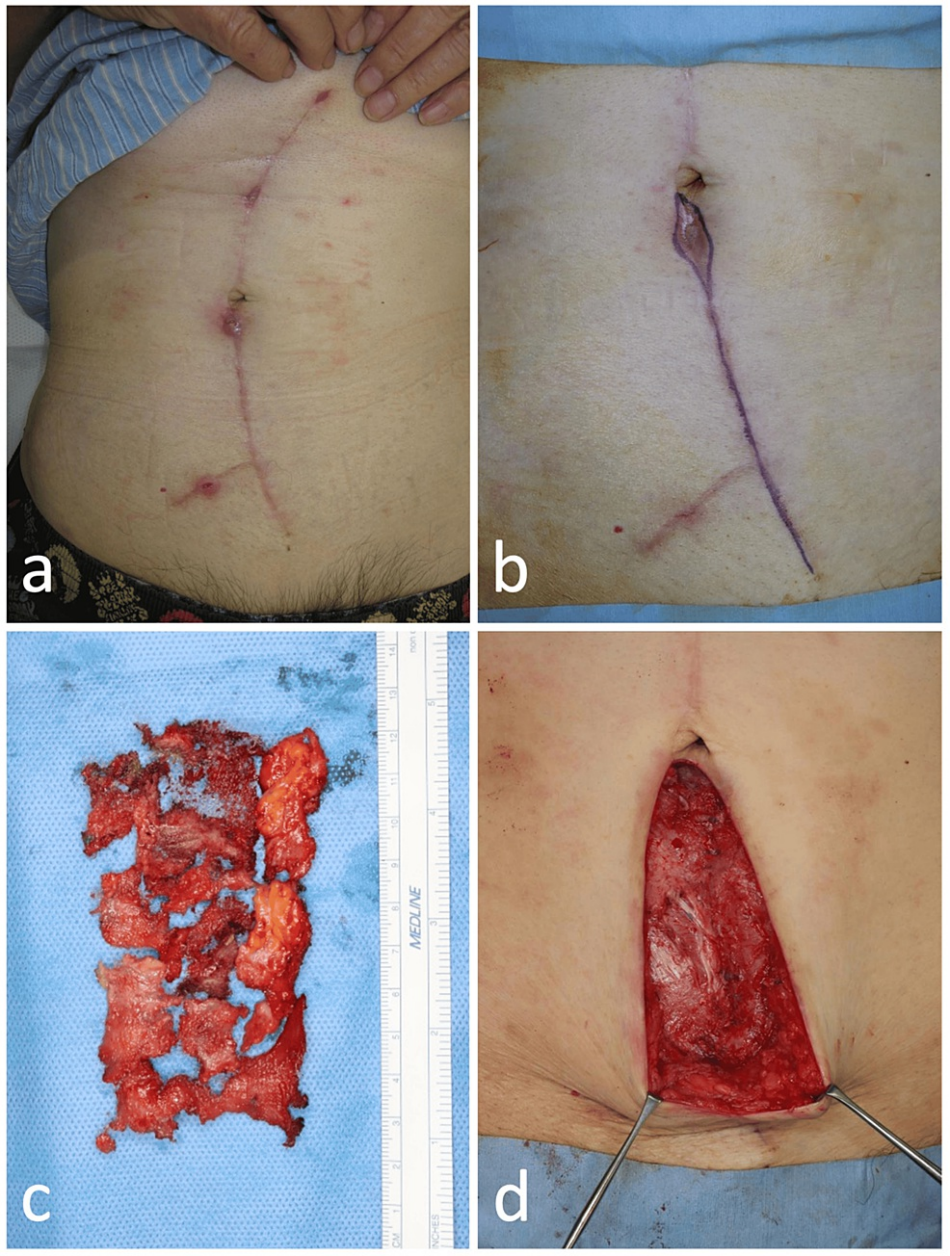
Abdominal wall mesh infection following harvesting of a rectus abdominis myocutaneous flap (case 1). Three months post-harvesting of a rectus abdominis myocutaneous flap for the reconstruction of a maxillectomy site, wound dehiscence was observed at the lower abdominal suture site (a). After two months of conservative treatment, excision of the fistula and removal of the mesh were performed (b). The excised polypropylene mesh, while fragmented, was completely removed within the visible range (c, d).

An 83-year-old male with right maxillary cancer underwent a total right maxillary resection, right cervical dissection, and maxillary reconstruction using a free rectus abdominis muscle flap. A mesh was fixed to the abdominal wall at the rectus abdominis muscle sampling site to prevent scar herniation. Approximately three months postoperatively, erythema was observed at the suture wound below the umbilicus at the time of rectus abdominis muscle extraction. Subsequently, the infection slowly expanded, and newly infected wounds and serous exudates appeared (Figure 1a). Although no causative organisms were detected in the general bacterial and fungal culture tests, and no elevated inflammatory response was observed in the blood draw (WBC count of 5,500/µL, CRP of 0.14 mg/dL), the clinical findings suggested a mild suture abscess or artifact infection (mesh infection), and the patient was treated with antibiotics (cephalexin) and povidone-iodine ointment. Conservative treatment failed to quell the infection, and because a hypoechoic area was observed subcutaneously under the ultrasound scan, wound debridement, including possible mesh removal, was performed under general anesthesia five months postoperatively for a subcutaneous abscess (Figures 1b-1d).

**Figure 2:**
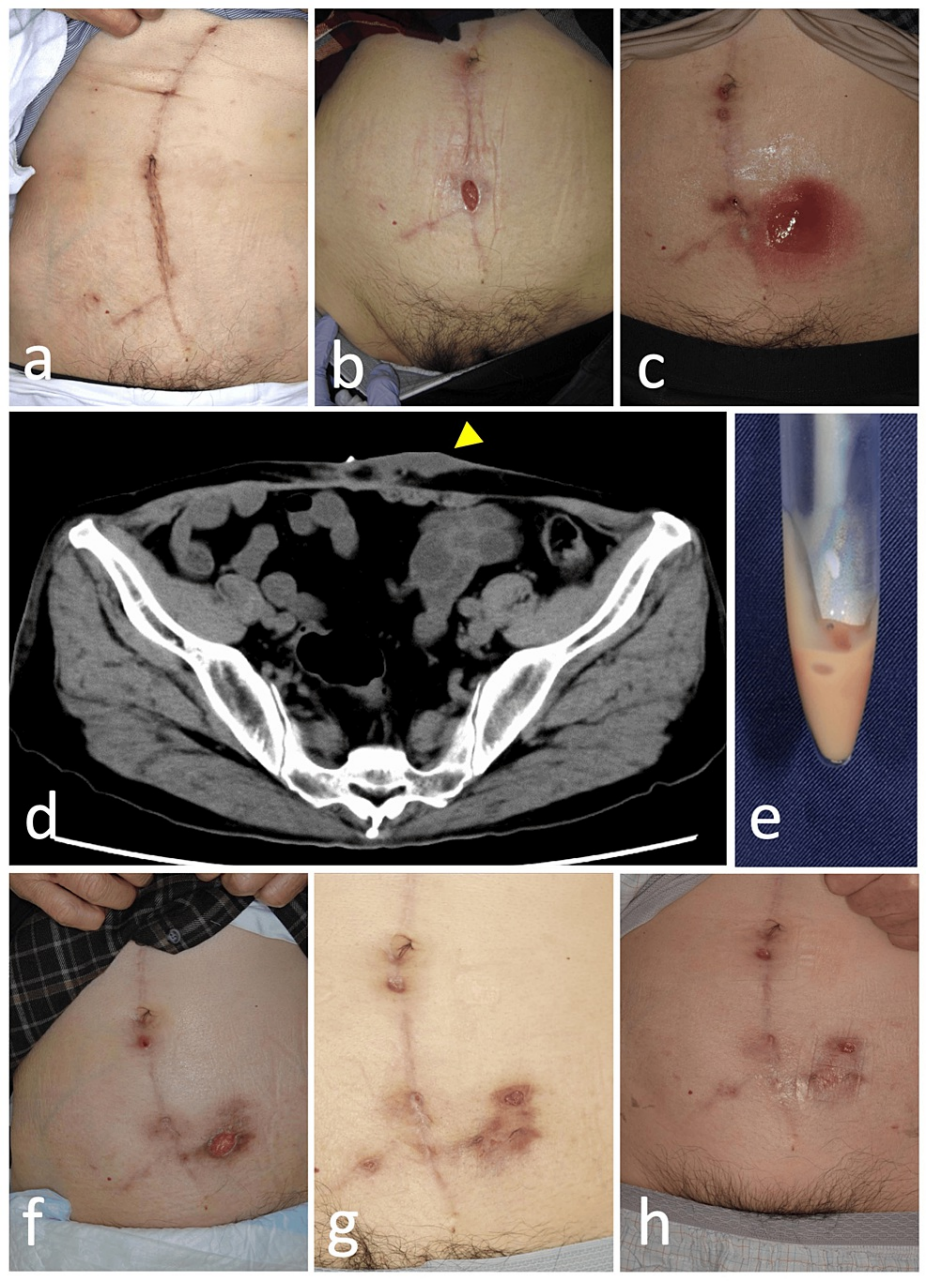
Recurrent mobilization of abdominal wall infection (continuation of case 1). No recurrence of infection was observed three weeks after mesh removal (a). Two months post-mesh removal, a skin ulcer with erythema developed (b). Despite local treatment with topical medications and oral antibiotics, a new, severely erythematous abscess formed four months after mesh removal (c). On the same day, a CT scan revealed a subcutaneous abscess (arrow head); aspiration yielded purulent fluid (e). Six months (f), eight months (g), and nine months (h) post-mesh removal, the ulcerated areas progressively expanded in a stepping-stone pattern.

The patient was discharged from the hospital without any postoperative signs of wound infection (Figure 2a). However, approximately two months after the mesh was removed, the suture wound below the umbilicus was separated, exposing bad granulation (Figure 2b). General bacterial and fungal cultures were submitted, but no causative organisms were detected, and blood samples still showed no significant elevation of the inflammatory response (WBC of 7,000/µL, CRP of 1.21 mg/dL). Although antibiotic (cephalexin) and povidone-iodine ointment treatment continued, the area of erythema expanded, leading to abscess formation four months after mesh removal (Figure 2c). The abscess formed a fistula, and computed tomography (CT) showed a subcutaneous abscess with a fistula connected to the skin on the body surface of the wound (Figure 2d). A drainage of the subcutaneous abscess was performed, and since an abscess was observed, it was subjected to a general bacterial culture; however, as in the previous case, the causative organism was not detected (Figure 2e). Six months after the initial debridement, povidone-iodine ointment treatment was continued, and although the fistula was constricted, complete epithelialization was not achieved (Figure 2f). Furthermore, eight months after the initial debridement, a new ulcer appeared around the suture wound (Figures 2g, 2h).

**Figure 3:**
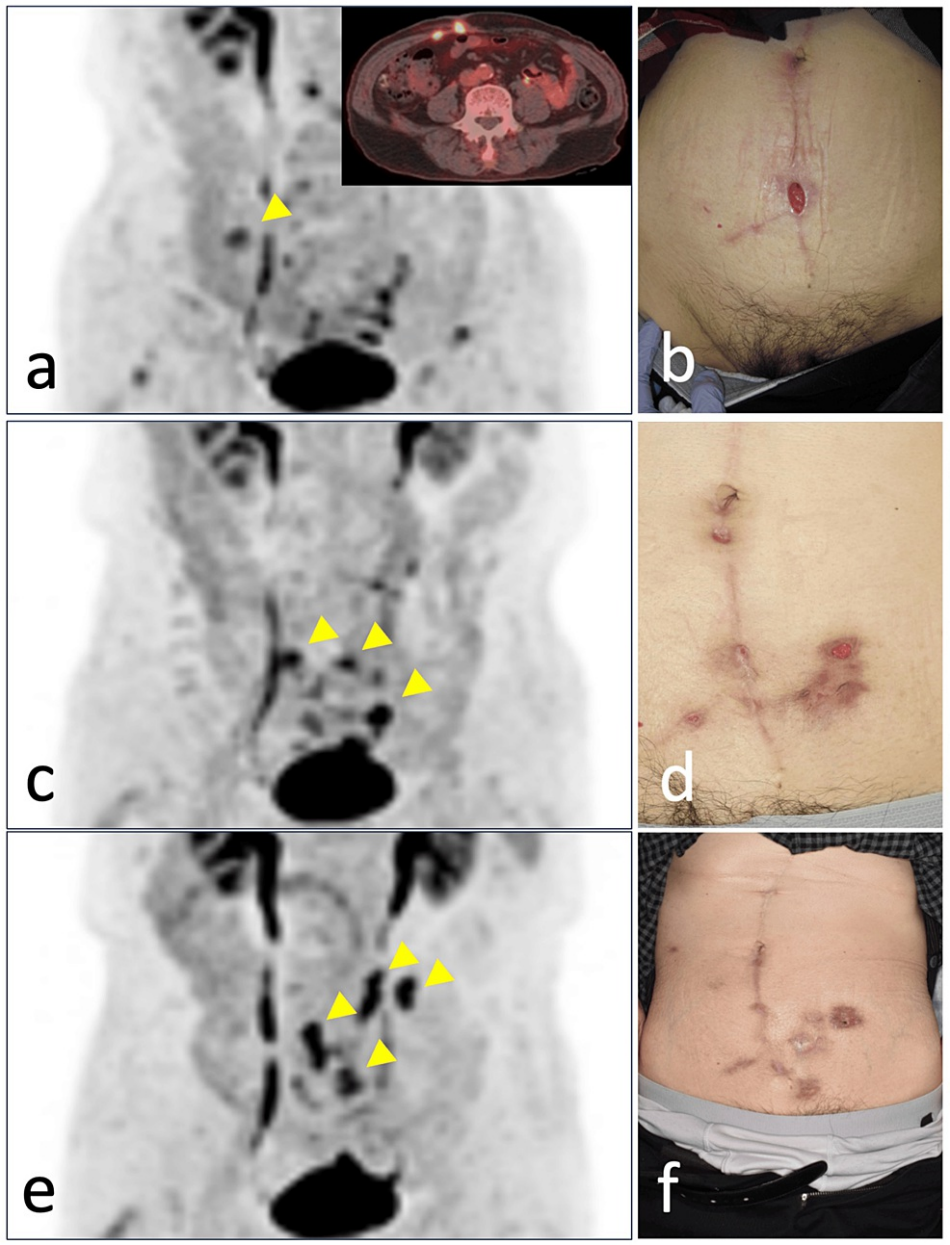
Assessment of infection locus migration via PET-CT (continuation of case 1). PET findings at six months (a), 12 months (c), and 18 months (e) post-maxillectomy for oral cancer. Arrowheads indicate regions where inflammation was suspected on PET. Clinical photographs of the abdominal wall at each respective time point are shown (b, d, f).

General bacterial and fungal culture tests were performed; however, no causative organism was detected, and the smear was negative. Positron emission tomography-computed tomography (PET-CT) was performed every six months to investigate the recurrence and metastasis of the maxillary carcinoma (Figures 3a-3f), and abnormal accumulation of [¹⁸F]fluorodeoxyglucose (FDG) was observed along the abdominal wall surgical wound. The initial diagnosis was considered a postoperative change, but the appearance of a new nodular shadow led us to suspect that it was due to recurrent metastasis of maxillary carcinoma.

**Figure 4:**
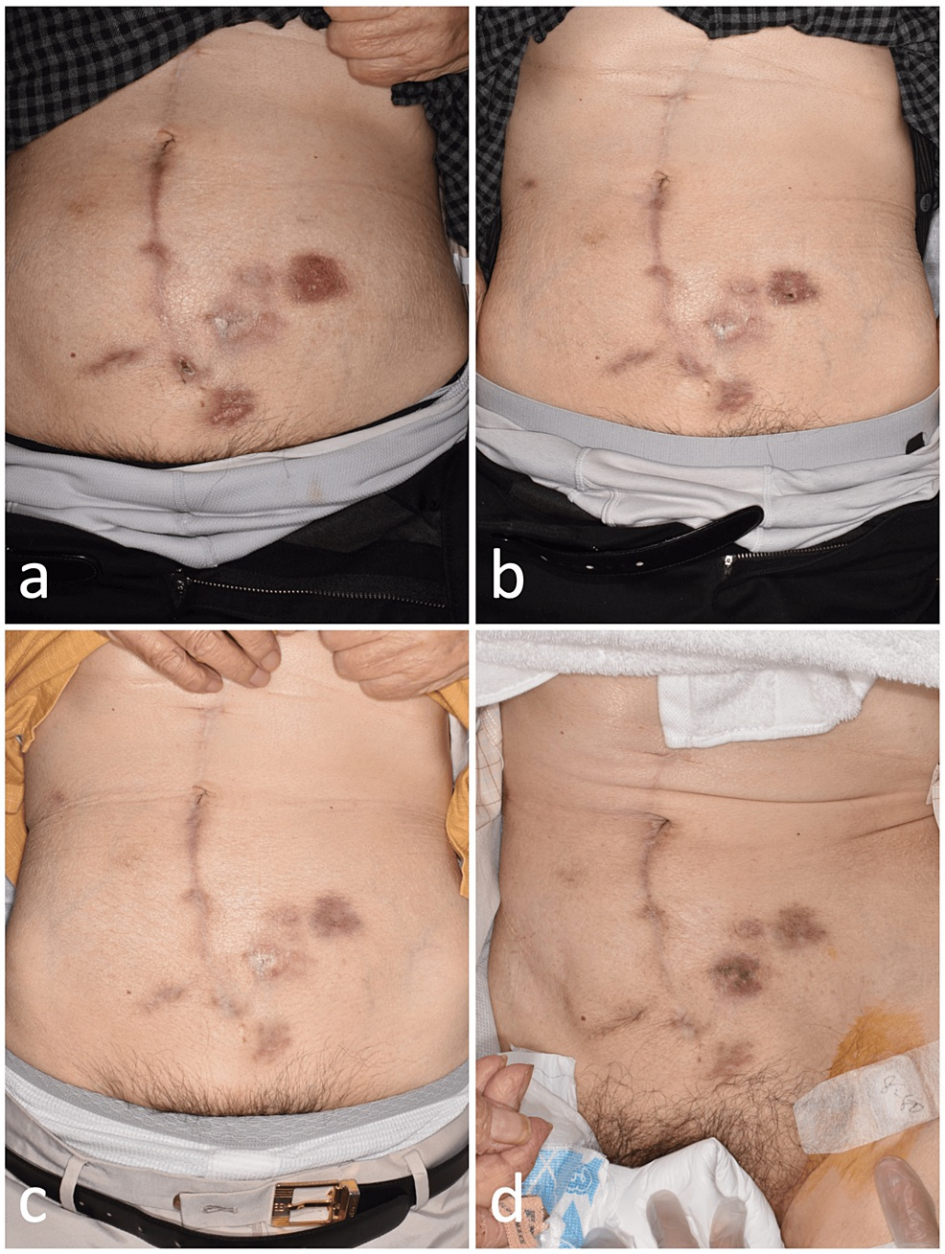
Progression after antibiotic treatment (continuation of case 1). Seventeen months after mesh removal, antibiotic treatment was initiated at this point (a). One month (b), three months (c), and five months (d) after starting antibiotics. The wound healed rapidly and no recurrence of infection was observed.

Nine months after the initial debridement, the abdominal wall fistula was removed, and additional debridement and wound closure of the surrounding necrotic tissue were performed (Figure 2h). Postoperatively, as in the previous debridement, the patient was discharged without signs of wound infection, and histopathological examination of the excised fistula was negative for neoplastic lesions. Three months after the second debridement, the patient showed erythema of the suture wound, which was thought to be an SSI, and the wound surgery team was consulted (Figure 4a). Mf was detected on an acid-fast culture test, leading to a diagnosis. After confirming the sensitivity, the patient was treated with a three-drug combination of moxifloxacin, clarithromycin, and Vibramycin for six months, and the wound ulcer improved without recurrence (Figures 4b-4d).

Case 2

**Figure 5:**
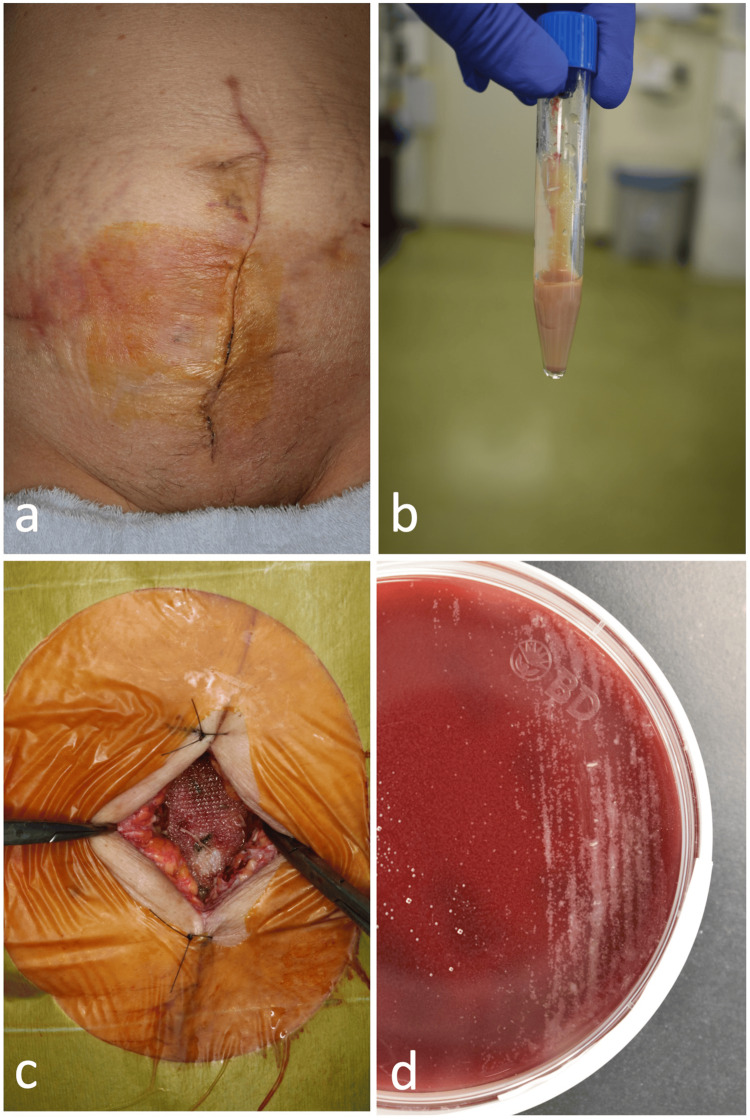
Abscess caused by Mycobacterium fortuitum after abdominal wall reconstruction for ventral hernia scar (case 2). Mild erythema was observed at three weeks post-abdominal wall reconstruction using polypropylene mesh for a ventral hernia scar (a). Aspiration yielded beige purulent fluid (b). A small incision was made for drainage and cleansing of the infected site (c). Rapid colony formation was observed upon culturing with blood agar medium (d).

A postoperative gynecological scar hernia was repaired by polypropylene mesh insertion in a 75-year-old female. Approximately three weeks postoperatively, the wound developed erythema with a wave-like sensation without heat (Figure 5a).

**Figure 6:**
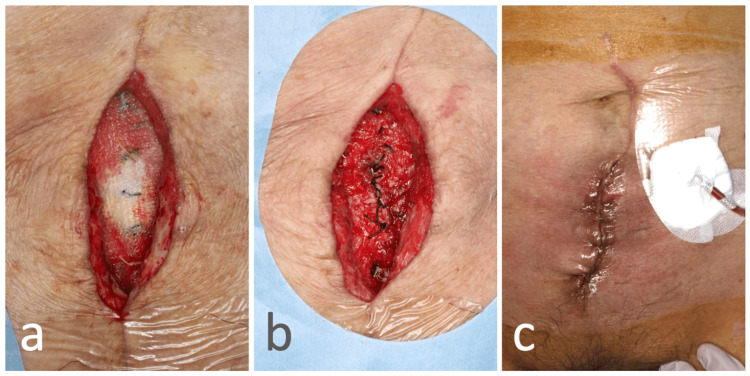
Debridement and closure of the abdominal wall (continuation of case 2). The mesh was removed one month after the abdominal wall scar hernia repair surgery: (a) before mesh removal and (b) after mesh removal. Following debridement, the wound was sutured closed (c).

Blood samples revealed no elevated inflammatory responses or systemic symptoms. In this case, the SSI also had a slow onset and differed from the usual SSIs. A puncture was performed on the erythematous area, and the pus was aspirated (Figure 5b). A small incision was made, and an abscess spread over the mesh was observed (Figure 5c). The patient was rinsed with a large amount of saline solution and underwent negative pressure wound therapy with continuous irrigation for three days; however, the infection could not be controlled. The patient was initially suspected of having an acid-fast bacterial infection because the general culture and smears were mildly positive on Gram staining (Figure 5d). Blood agar medium was added, and rapid growth was observed, leading to a diagnosis of atypical antimicrobial infection. Furthermore, Mf was detected. One week after the onset of the SSI, the mesh was removed, the wound was sutured closed, and the patient underwent three months of anti-acid therapy (levofloxacin) without recurrence (Figures 6a-6c).

In case 1, it took approximately 17 months to detect and initiate treatment with acid-fast bacteria. Conservative ointment treatment, empiric antibiotic therapy, and surgical excision were performed. However, the lesion recurred and was difficult to treat. In case 2, acid-fast bacilli were detected at an early stage, and prompt antibiotic and surgical treatment were performed, which resulted in prompt treatment.

## Discussion

*Mycobacterium tuberculosis*, NTM, and special nutrient-demanding mycobacteria are the major groups of acid-fast bacilli. *Mycobacterium tuberculosis* includes *M. tuberculosis* and *M. bovis*, whereas non-tuberculous *Mycobacterium tuberculosis* is classified into slow-growing mycobacteria (SGM), such as *M. avium* complex (MAC) and *M. kansasii*, and rapid-growing mycobacteria (RGM) that form colonies within seven days after the start of culture. RGM form colonies within seven days of culture initiation. Specific nutrient-demanding bacteria include *M. leprae*. Mf is a non-tuberculous RGM belonging to Runyon class IV [7] that is widely distributed in nature, such as in soil and water [8]. Furthermore, *M. marinum* and *M. chelonei *are the most frequently reported causative organisms of cutaneous non-tuberculous Mycobacterium tuberculosis infections, followed by MAC, *M. kansasii*, and *M. abscessus* [11]. Mf is rarely the causative agent of pulmonary infections among RGM and has been reported to cause skin and soft tissue infections, bone and joint infections, and systemic disseminated infections. Mf has also been reported to cause bloodstream and SSIs, and SSIs caused by Mf account for 60% to 80% of the SSIs caused by RGM [12]. Both patients had risk factors such as diabetes for SSI. In addition, case 1 worked in the freshwater fish industry, and case 2 was a farmer who were assumed to be infected with Mf in their living environment. Skin and soft tissue infections with RGM have been reported in the United States, Taiwan, India, and other regions [12]. Bacterial culture is useful in the diagnosis of SSI by Mf, but it usually takes five to seven days or more from the culture to detect the bacteria, which is slower than the detection of general bacteria. It has been noted that by the time Mf is detected, the culture may have been discarded or judged negative [13]. SSIs caused by Mf tend to lack characteristic clinical symptoms, and it is difficult to reach a diagnosis unless an antimicrobial culture is suspected or a two-week general bacterial culture is performed. Although there have been few reports of SSI caused by Mf in Japan, we believe that there are a small number of cases that have not been diagnosed as false negatives.

NTM infections cause systemic symptoms in immunocompromised individuals. In contrast, in immunocompetent individuals, NTM infections often cause localized infections, such as skin and soft tissue infections, without symptoms, such as fever and chills. Among NTM, infections caused by RGM are common, especially in medical-related and postoperative skin and soft tissue infections. The clinical manifestations of RGM skin and soft tissue infections include cellulitis, abscess formation, fistula formation, and SSI, which often develop one month postoperatively and are often followed by local findings after discharge. Similarly, Mf infections have been reported in immunocompromised individuals, such as chemotherapy and long-term steroid users [14]. Furthermore, the frequency of lung and disseminated disease is low, and skin infections in immunocompromised individuals have been reported frequently. Diagnosis is often difficult owing to the lack of characteristic findings, as the infection is often localized rather than systemic. When chronic inflammation or abscess formation occurs postoperatively, empiric antibiotics are often administered for SSI; however, if there is a poor response to treatment and smears and bacterial cultures are negative, RGM (especially Mf) should be suspected among NTM.

In our case, the time to the onset of infection was after the postoperative period (three to 12 weeks). In both cases, there were no systemic symptoms or inflammatory findings, such as a cold abscess, and the infected wounds developed relatively gently and continued to form abscesses. Although both patients had typical NTM infections, we judged that case 1 was an ordinary bacterial infection and treated it with empiric antibiotic therapy and mesh removal without considering NTM infection as a differential diagnosis because of the lack of reports in Japan. Non-susceptible antibiotic therapy and incomplete removal of the mesh, which caused scarring due to its prolonged course, failed to quell the infection, and new abscesses continued to appear postoperatively with intradermal metastasis. While normal prosthetic infections often improve with the removal of the prosthesis and antibiotic treatment, Mf infections spread not only to the prosthesis but also to the surrounding soft tissues, which may have been the reason for the difficulty in treatment. This process was considered to be due to infection and metastasis within the soft tissues. Surgical treatment alone was not sufficient to completely remove the infected wound, as the abnormal FDG accumulation sites repeatedly disappeared and appeared over time on PET-CT. No previous PET-CT scans of SSI due to Mf were found in the literature, and this is a very interesting finding that indicates intradermal metastasis. If Mf infection is suspected, early removal of the artifact and treatment with antimicrobials, as in case 2, will result in a rapid course of treatment without intradermal metastasis.

The usefulness of histopathological examinations in the diagnosis of NTM infections has also been reported. It has been shown that NTM infection does not always present with dry necrosis on histopathology [15], and no characteristic pathology was seen in case 1. Although some of the specimens submitted at the time of the second debridement showed multinucleated giant cells of the foreign body type, there were no Langhans-type giant cells, which are common in NTM infection, nor were there any findings suggestive of xerophilic necrosis. After NTM infection was determined, Ziehl-Neelsen staining of the specimen was performed, but there were no findings suggestive of acid-fast bacillus infection. Ziehl-Neelsen staining is not necessarily positive in NTM infections, and histopathological examination alone is not sufficient to exclude infection with NTM. We believe that histopathological examination alone is insufficient to rule out infection with NTM.

Effective treatment of RGM infections involves appropriate antibiotic and surgical treatment. It has been reported that surgical treatment alone often results in recurrence within four to six weeks after surgery [10], which we believe is due to the metastasis of Mf to the surrounding soft tissues, as described above. Therefore, it is important to check the susceptibility to Mf and the administration of antibiotics. Although there are no clear criteria for the selection and duration of antibiotic therapy appropriate for the NTM infection itself [10], skin and soft tissue infections due to Mf should be treated with at least two susceptible agents for at least six months [12].

The antibiotic of choice for Mf infection is generally administered after confirming the susceptibility. Sometimes, Mf can be resistant to standard antituberculosis drugs. However, Mf has been reported to exhibit susceptibility to antibiotics such as amikacin (AMK; aminoglycoside), CPFX (cyclofloxacin: new quinolone), OFLX (ofloxacin: new quinolone), IPM/CS (imipenem/cilastatin: carbapenem), and CAM (clarithromycin: macrolide) [16], which are highly susceptible to new quinolones [17].

Regarding mesh infection due to Mf, no consensus has been reached on whether the mesh should be removed. Mesh infection is usually treated with antibiotics first, and then wound opening and cleaning therapy are attempted, and mesh removal is considered if the wound infection persists [18]. Mf infections often develop late, leading to deep infection. Considering the biofilm-forming nature of the infection [7], mesh removal is often performed [19] without quiescence of the infection by incisional drainage or washing alone. There are limited reports on the removal of prosthetic infections other than mesh infections, such as breast implants and artificial joints after conservative treatment and continuous washing, which failed to control the infection. We believe that local cleansing and antibiotics alone may not be sufficient to quell Mf infection because Mf infection forms biofilms, and intradermal metastasis may occur over a long period of time, as shown in the PET-CT of case 1. It is important to detect Mf at an early stage and initiate surgical treatment with sensitive antibiotics. It has been suggested that the treatment of Mf infection should be combined with appropriate multidrug therapy and early surgical treatment. In cases where Mf causes an artifact infection, long-term antibiotic therapy, in addition to artifact removal, has been reported [20]. In the case of Mf with artifact infection, long-term antibiotic therapy is required in addition to the removal of the artifact. If a patient presents with a slow, localized SSI approximately one month after surgery, is unresponsive to antibiotic therapy, and has a negative bacterial culture, aggressive suspicion and detection of NTM infection are important to initiate treatment at an early stage and obtain a prompt cure.

## Conclusions

We encountered two cases of mesh infection from an SSI caused by Mf after the abdominal wall was reinforced with an artificial mesh. NTM infection (especially Mf infection) should be suspected in SSI with a slow postoperative appearance, poor response to empirical antibiotics, conservative or surgical treatment, and a negative general bacterial culture. The patients showed a different disease course owing to the different timing of the intervention. It is important to start treatment at an early stage because PET/CT has shown that Mf infection can cause intradermal metastasis over a long-term course. We believe that surgical mesh removal alone is not sufficient for treatment and that the use of susceptible antimicrobial agents will accurately induce sedation of the infection. Therefore, treatment may be difficult if NTM infection is not considered a differential cause of postoperative SSI.
